# Knowledge of stroke risk factors and warning symptoms among adults with type 2 diabetes in Addis Ababa, Ethiopia, 2021: an institution-Based cross-sectional study

**DOI:** 10.1186/s12872-022-03031-8

**Published:** 2023-01-16

**Authors:** Rediet Akele Getu, Fekadu Aga, Tadesse Badada, Sewnet Getaye Workie, Makda Abate Belew, Kalkidan MekonnenRN

**Affiliations:** 1 Department of Nursing, School of Nursing and Midwifery, College of Health Sciences, Debre Berhan University, Debre Berhan, Ethiopia; 2 School of Nursing and Midwifery, College of Health Sciences, Addis Ababa University, Addis Ababa, Ethiopia; 3 Department of Public Health, School of Public Health, College of Health Sciences, Debre Berhan University, Debre Berhan, Ethiopia

**Keywords:** Knowledge, Stroke, Risk factors, Warning symptoms

## Abstract

**Background:**

Stroke is a global public health concern with type 2 diabetes being one of the common risk factors. Knowledge of stroke risk factors and warning symptoms among type 2 diabetes patients is largely unknown in developing countries like Ethiopia. The inability to recognize stroke warning symptoms accurately is an important cause of delay in seeking medical attention. We investigated knowledge of stroke risk factors and warning symptoms among adults with type 2 diabetes and the factors associated with these variables.

**Methods:**

This was an institution-based cross-sectional study. We enrolled 470 adult type 2 diabetes patients using a systematic random sampling method from four government-managed hospitals in Addis Ababa from 1 to 30 February 2021. The Stroke Recognition Questionnaire was adapted to measure the knowledge of stroke risk factors and warning symptoms. Sociodemographic characteristics, source of information, and reaction to stroke were also measured. Data were analyzed using SPSS version 25 and linear regression analysis was used to determine factors independently associated with knowledge of stroke risk factors and warning symptoms.

**Result:**

The mean age of the participants was 50.6 ± 12.9 years. The mean score of knowledge of stroke risk factors and warning symptoms was 67.2% and 63.9%, respectively. Higher educational level (B = 2.007, 95% CI = 1.101, 2.914, *P* < 0.001), knowing someone diagnosed with stroke (B = 3.328, 95% CI = 2.734, 3.922, *P* < 0.001), and living with others (B = 2.28, 95% CI = 1.606, 2.954, *P* < 0.001) were independently associated with knowledge of stroke risk factors. Younger age (B = − 0.021, 95% CI= -0.038, 0.005, *P* = 0.01), higher educational level (B = 1.873, 95% CI = 1.017, 2.730, *P* < 0.001), and knowing someone diagnosed with stroke (B = 3.64, 95% CI = 3.079, 4.200, *P* < 0.001) were independently associated with knowledge of warning symptoms of stroke.

**Conclusion:**

The mean score of knowledge of stroke risk factors and warning symptoms was 67.2% and 63.9%, respectively. Younger age, higher educational level, and living with other people are predictors of better knowledge of stroke risk factors and warning symptoms in this study. Future educational interventions should target type 2 diabetes adults with advancing age and the involvement of their family members.

**Supplementary Information:**

The online version contains supplementary material available at 10.1186/s12872-022-03031-8.

## Background

Stroke is one of the common cerebrovascular disorders characterized by an acute clinical episode of focal or global neurological disturbance associated with impairment of cerebral circulation [1]. The burden of stroke is an enormous public health concern worldwide. It is a leading cause of mortality and disability, especially in low-income and middle-income countries [2]. The Global Burden of Disease Study (GBD) 2019 reported that stroke is the second-leading cause of death and the third-leading cause of death and disability combined worldwide [3]. The same source indicates that age-standardized stroke-related death and disability rates are significantly higher in low-income countries than in high-income countries. Studies have shown that sub-Saharan Africa has high age-standardized stroke incidence and prevalence rates [4] and higher case fatality [5–7]. In Ethiopia, stroke is among the top three prevalent cardiovascular diseases (CVD) and the second leading cause of CVD deaths [8]. The pooled burden of stroke is 46.42% for hemorrhagic and 51.40% for ischemic stroke in Ethiopia [9]. A prospective study also reported 57.1% incidence of ischemic and 34.5% hemorrhagic stroke in Ethiopia [10]. Studies have shown that about two-thirds of hospitalized stroke patients in this country have poor treatment outcomes [11] and 18% die during hospitalization [12].

Studies have documented several risk factors and symptoms of a stroke. The major modifiable risk factors of stroke are hypertension, cigarette smoking, diabetes, obesity, poor diet, physical inactivity, heart failure, arterial fibrillation, excessive alcohol consumption, and high blood cholesterol [9, 10, 13–19]. The symptoms of stroke include numbness or weakness on one side of the body, confusion or trouble speaking or difficulty understanding speech, trouble walking or loss of body balance, trouble seeing, and sudden severe headache [19]. The modifiable risk factors are responsible for 90% of the global burden of stroke [14, 20] with hypertension being the major causal risk factor for the incidence and outcome of stroke [20]. These established risk factors account for a larger part of stroke both in young and older adults with some degree of variations [21, 22].

Type 2 diabetes is among the well-established yet modifiable risk factors of stroke [23, 24]. Individuals with type 2 diabetes have a 1.5 to 3 times increased risk of stroke than those without diabetes [25]. A large meta-analysis study revealed that diabetes is responsible for 2.27 times increased risk of developing ischemic stroke and 1.56 times for hemorrhagic stroke [26]. Diabetes is also associated with an increased risk of stroke recurrence in persons with ischemic stroke [27]. There is a sex disparity in the risk of diabetes-related stroke, with women having a 2.3 times increased risk while men have 1.8 times increased risk of stroke [28]. Diabetic patients are also at higher risk of suffering from hypertension, myocardial infarction, and high cholesterol [23] which may further increase their likely hood of developing stroke.

Knowledge of stroke risk factors and warning symptoms is essential for the prevention and treatment of stroke among high-risk individuals such as those with type 2 diabetes. Thus, understanding patients’ knowledge of stroke risk factors and symptoms is essential for designing and implementing interventions that improve help-seeking behavior and reduce pre-hospital delay [29–31]. Improving the awareness of stroke risk factors and the seriousness of symptoms facilitates timely diagnosis and maximizes therapeutic outcomes. This is particularly important in settings like Ethiopia where pre-hospital delay [32] and in-hospital mortality [12] for stroke are substantially high. However, there is a dearth of research evidence in Ethiopia concerning the knowledge of stroke risk factors and symptoms among type 2 diabetic patients. Therefore, the purpose of this study is describing the knowledge of stroke risk factors and warning symptoms in patients with type 2 diabetes with emphasis on factors associated with it.

## Methods and materials

### Design and participants

An institution-based cross-sectional analytic study was conducted among adults with type 2 diabetes in four government-managed tertiary hospitals in Addis Ababa using a proportionally allotted stratified sampling method from 1 to 30 February 2021. The source population was all type II diabetic patients who were on follow-up at governmental hospitals in Addis Ababa. All type II diabetes patients attending the diabetic clinic at TikurAnbessa, Menelik II, Zewditu memorial, and Yekatit 12 hospitals were considered as the study population and each individual with type II DM attending a diabetic clinic in those selected hospitals during the study period was considered as a study unit. The study followed the fundamental principles of research ethics and was approved by the Institutional Review Board of the College of Health Sciences at Addis Ababa University. Authorities in the study settings granted permission to conduct the research and informed consent were obtained from each participant. A total of 499 sample size was calculated using a single population proportion formula with the assumption of a 95% confidence interval, 5% margin of error, and 18.3% proportion of patients with good knowledge of stroke risk factors in a previous study [33], a design effect of 2, and 10% non-response rate. The study participants were enrolled using a systematic sampling method with patients’ record order in the follow-up appointment list serving as a sampling frame. Every 5th patient on the record was enrolled from each hospital with the first one selected using a lottery method. The inclusion criteria were being a type 2 diabetes patient on follow-up at least for the last 6 months; 18 years or older; and can communicate in the Amharic language. The exclusion criteria involved having a confirmed diagnosis of stroke, critical illness, severe mental illness, and inability to communicate independently. The interviewer-administered questionnaire was completed immediately after the participants provided consent.

### Sampling procedure


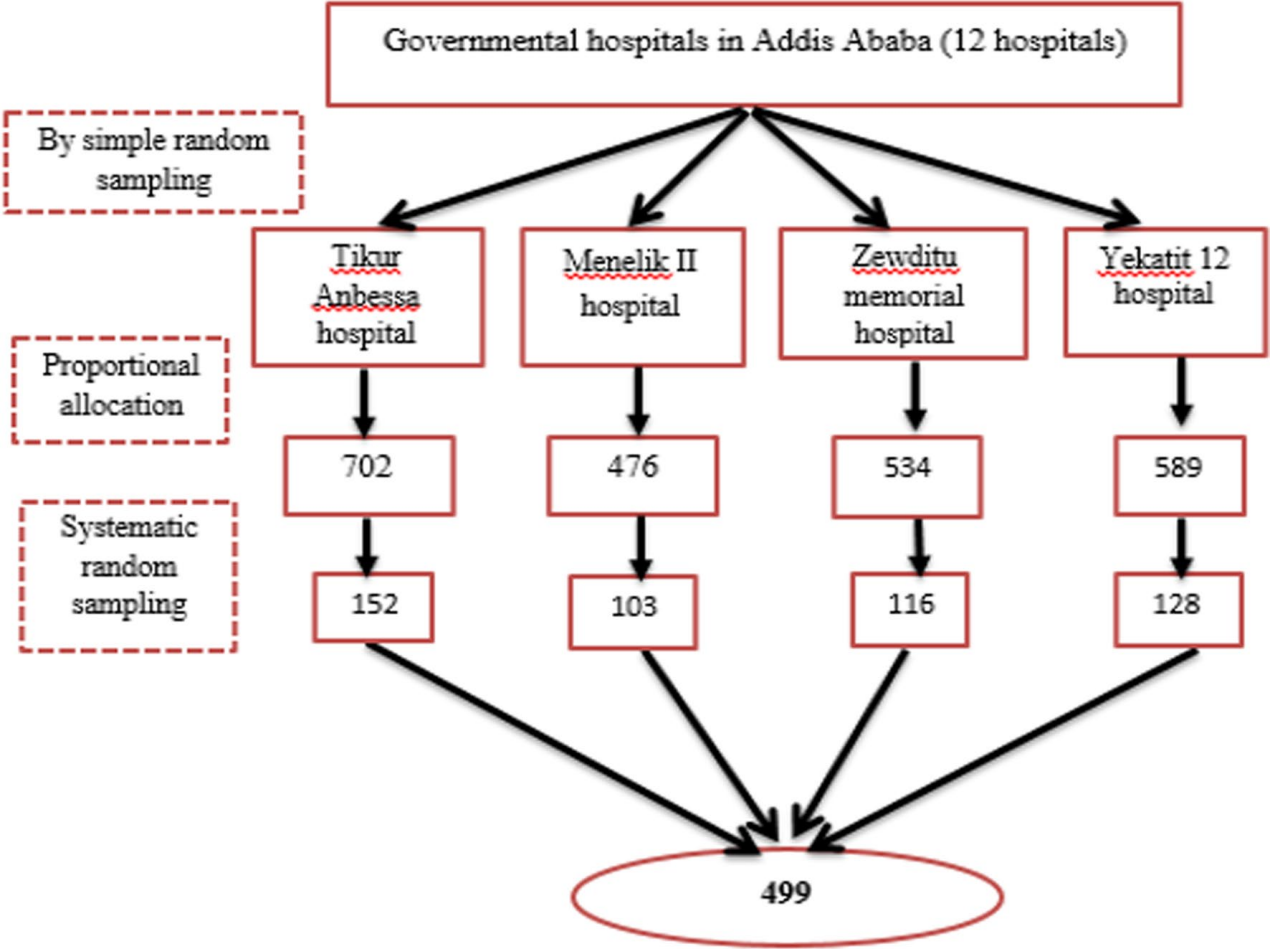


### Data collection methods and instrument

Data were collected using an interviewer-administered questionnaire. The data was collected by 4 BSc nurses who were properly trained for three days about the instrument and ways of approaching the participants and how to obtain consent before data collection. The interviewer-administered questionnaire consisted of 5 parts: socio-demographic characteristics, knowledge of stroke risk factors, knowledge of stroke warning symptoms, source of information for stroke, and reaction to stroke. The interviewer-administered questionnaire was first translated from English to Amharic language and backward translated to English using two independent bilingual translators to ensure the accuracy of the Amharic version of the questionnaire. The interviewer-administered questionnaire was pretested on 5% of the sample size (type 2 diabetes patients) in a hospital which was not part of the main study settings. The pretest data were used to evaluate the questionnaire for clarity and cultural acceptability.

Socio-demographic characteristics such as age, sex, marital status, income, and education were collected using seven structured items included in the interviewer-administered questionnaire. The source of information for stroke was measured using three structured items in which one item inquired whether the participant ever heard about a stroke, the second item asked those who responded ‘yes’ to the first item from where they got the information, and the third item asking if the participant had known somebody diagnosed with stroke. Reaction to stroke was assessed using a single item with six options such as calling an ambulance, driving the patient to a religious leader, giving a drink or food, waiting until recovery, trying to calm the person, and don’t know what to do.

The ***Stroke Recognition Questionnaire (SRQ)*** was adapted to measure the knowledge of stroke risk factors and warning symptoms [19]. The SRQ consists of 10 items for the knowledge of stroke risk factors subscale and 10 items for the knowledge of stroke warning symptom subscale. In the current study, 2 items were added to the knowledge of stroke risk factors part, making it a 12-item subscale. The items in both subscales were dichotomous (Yes/No). For both stroke risk factors and warning symptoms subscales, knowledge was scored by the proportion of correctly answered items. The total scores range from 0 to 100 in both subscales. The Cronbach’s alpha reliability for the stroke risk factors subscale was 0.70 and that of stroke warning symptoms was 0.81 in the previous study [19]. The Cronbach’s alpha in our study was 0.79 for the stroke risk factors subscale and 0.83 for the stroke warning symptoms subscale.

### Statistical analysis

All data were coded and entered into Epi Data software version 4.6.0 for cleaning and then exported to SPSS for Windows software version 25 for analysis. Descriptive statistics including frequency, percentage, mean and standard deviations were used to summarize the data. Linear regression analysis was used to identify factors independently associated with the outcome (dependent) variables – knowledge of stroke risk factors and warning symptoms. The assumption of data normality was checked using Kolmogorov-Smirnov and Shapiro-Wilk tests, histograms, and Q-Q plots and confirmed that not violated. Variance inflation factors (VIF) and tolerance tests were used to ensure the assumption of multicollinearity was met. Multi-categorical independent (predictor) variables were dummy coded before running the regression analysis. Simple and multiple linear regression analyses were used to show the association between independent and dependent variables. All theoretically relevant independent variables were entered into the regression models. Those variables whose *P*-value < 0.25 in simple linear regression were entered into multiple linear regression analysis and statistical significance was declared using a 95% confidence interval and *P*-value < 0.05 (Additional file 1).

## Results

### Socio-demographic characteristics

A total of 470 type 2 diabetes patients participated in this study with a response rate of 94.2%. Table 1 show that the mean age of the participants was 50.6 ± 12.9 years. More than half of the participants (54.9%) were female, the majority of them (83%) lived with others, and most of them (38.7%) completed college/university education.

**Table 1:** Sociodemographic characteristics of the study participants (N = 470)

Variable	Frequency (n)	Percentage (%)
Age in years, Mean ± SD	50.6 ± 12.9	
Sex		
Male	212	45.1
Female	258	54.9
Marital status		
Single	88	18.7
Married	345	73.4
Divorced	16	3.4
Income	21	4.5
< 2250 ETB	206	43.8
2250–8900 ETB	214	45.5
> 8900 ETB	50	10.6
Educational status		
Do not read and write	37	7.9
Read and write	41	8.7
Primary school	73	15.5
Secondary school	137	29.1
College/ University	182	38.7
Occupation		
Government employer	110	23.4
Merchant	45	9.6
Housewife	92	19.6
Student	5	1.1
Daily laborer	16	3.4
Farmer	4	0.9
Retired	41	8.7
Self-employed	127	27
Unemployed	30	6.4
Living situation		
Living alone	80	17
Living with others	390	83

*SD*  standard deviation, *ETB* Ethiopia Birr (currency)

### Knowledge of stroke risk factors

We have calculated the composite or total score of the correctly answered items in percent. Accordingly, the mean score of knowledge of stroke risk factors was 67.2%±17.1%. Table 2 shows the individual risk factors identified by the study participants. Most of the participants identified hypertension (90.4%), obesity (87.2%), stress (85.5%), lack of physical activity (85.3%), and alcohol (84%) as risk factors for stroke.

**Table 2:** Stroke risk factors identified by type 2 diabetes patients (N = 470)

Risk factor	Response	Frequency	Percent
Hypertension	Yes	425	90.4
	No	45	9.6
Diabetes	Yes	373	79.4
	No	97	20.7
High blood cholesterol	Yes	376	80.0
	No	94	20.0
Smoking	Yes	401	85.3
	No	69	14.7
Alcohol	Yes	395	84.0
	No	75	15.9
Obesity	Yes	410	87.2
	No	60	12.7
Lack of physical activity	Yes	401	85.3
	No	69	14.7
History of having a heart attack	Yes	345	73.4
	No	125	26.6
Irregular heartbeat	Yes	387	82.3
	No	83	17.7
History of neck vein disease	Yes	327	69.6
	No	143	30.4
Stress	Yes	402	85.5
	No	68	14.4
Older age	Yes	299	63.6
	No	171	36.4

### Knowledge of stroke warning symptoms

We have calculated the composite or total score of the correctly answered items in percent. Thus, the mean score of knowledge of stroke warning symptoms was 63.9 ± 15.4%. Table 3 shows the individual stroke warning symptoms identified by the study participants. The majority of the participants identified trouble in body coordination (92.1%), followed by sudden unilateral numbness/weakness of the body (90.9%), trouble walking (89.6), sudden unexplained dizziness (87.0%), and loss of balance (85.1%).

**Table 3:** Stroke warning symptoms identified by type 2 diabetes patients (N = 470)

Symptoms	Response	Frequency	Percent
Slurred speech	Yes	362	77.0
	No	108	23.0
Numbness on one side of the face	Yes	392	83.4
	No	78	16.6
Weakness on one side of the body	Yes	427	90.9
	No	43	9.1
Trouble walking	Yes	421	89.6
	No	49	10.4
Loss of balance	Yes	400	85.1
	No	70	14.9
Trouble with coordination	Yes	433	92.1
	No	37	7.9
Confusion	Yes	328	69.8
	No	142	30.2
Sudden unexplained dizziness	Yes	409	87.0
	No	61	13.0
Double vision	Yes	370	78.7
	No	100	21.3
Sudden severe headache	Yes	390	83.0
	No	80	17.0

### Source of stroke information

**Fig. 1:**
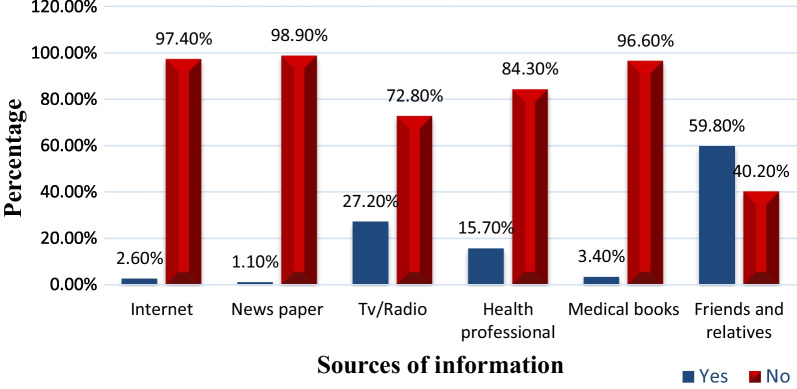
Source of stroke information among type 2 diabetes patients (N = 470)

Figure 1 shows that more than half of the participants (59.8%) heard about stroke from friends and relatives followed by TV and radio (27.2%). Only 15.7% of the participants had received information about stroke from health professionals.

### Reaction to stroke symptoms

**Fig. 2:**
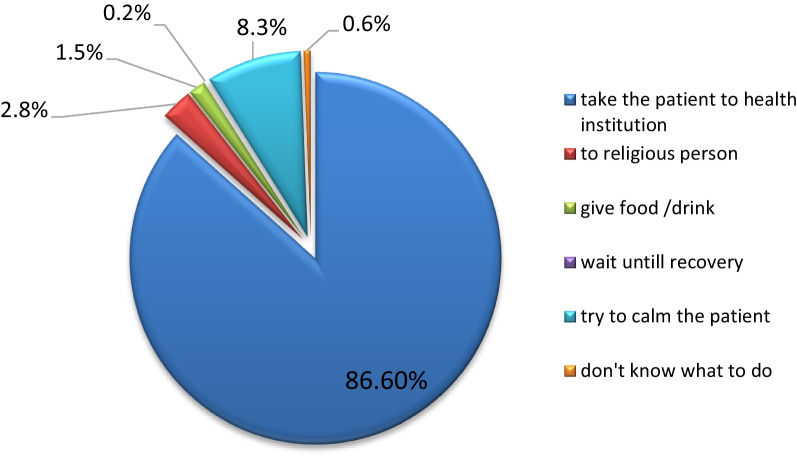
Reaction to stroke symptoms among type 2 diabetes patients (N = 470)

Figure 2 shows the study participant’s reaction when coming across somebody with stroke symptoms. The majority of them (86.6%) responded that they would take the person to the health facility followed by 8.3% try to calm the person with stroke symptoms.

### Factors associated with knowledge of stroke risk factors

Multiple linear regression analysis (Table 4) revealed that participants who completed college/university education had an increased knowledge score of stroke risk factors by a factor of 2 compared with other educational categories (B = 2.007, 95% CI = 1.101, 2.914, *P* < 0.001). Participants who live with other people had an increased knowledge score of stroke risk factors by a factor of 2.2 compared with participants who live alone (B = 2.28, 95% CI = 1.606, 2.954, *P* < 0.001). Participants who knew someone diagnosed with stroke had an increased knowledge score of stroke risk factors by a factor of 3.3 compared to those who did not know (B = 3.328, 95% CI = 2.734, 3.922, *P* < 0.001).

**Table 4:** Multiple linear regression analysis of factors associated with knowledge of stroke risk factors among type 2 diabetes patients (N = 470)

Variable	B	SE of B	Adj.β.coff	*t*-test	*P*	95% CI for B	Collinearity statistics	
							Tolerance	VIF
Level of education								
Read and write	1.039	0.522	0.091	1.989	0.047	(0.012, 2.065)	0.51	1.96
Primary school	0.596	0.471	0.067	1.264	0.207	(− 0.33,1.522)	0.38	2.63
Secondary school	1.158	0.456	0.163	2.54	0.011	(0.262,2.054)	0.26	3.87
College/ University	2.007	0.461	0.302	4.351	**0.000**	(1.101,2.914)	0.22	4.56
*Living situation*								
Living with others	2.28	0.343	0.265	6.645	**0.000**	(1.606,2.954)	0.66	1.5
*Know someone with a stroke (Yes)*	3.328	0.302	0.422	11.02	**0.000**	(2.734,3.922)	0.72	1.39

### Factors associated with stroke warning symptoms

The three variables significantly associated with knowledge of stroke warning symptoms were age, college/university education, and knowing someone diagnosed with stroke. Table 5 shows that participants who completed college/university education had an increased knowledge score of stroke warning symptoms by a factor of 1.8 compared to those in other educational categories (B = 1.873, 95% CI = 1.017, 2.730, *P* < 0.001). Likewise, participants who knew someone diagnosed with stroke had an increased knowledge score of stroke warning symptoms by a factor of 3.6 compared to those who did not know (B = 3.64, 95% CI = 3.079, 4.200, *P* < 0.001). Finally, for every-one year increase in age knowledge score of stroke warning symptom decreases by 2% (B = − 0.021, 95% CI = − 0.038, 0.005, *P* = 0.01).

**Table 5:** Multiple linear regression analysis of factors associated with knowledge of warning symptoms of stroke among type 2 diabetes patients (N = 470)

Variable	B	SE of B	Adj.β.coff	*t*-test	*P*-vale	95% CI for B	Collinearity statistics	
							Tolerance	VIF
*Age*	− 0.021	0.008	− 0.09	− 2.603	**0.01**	− 0.038, − 0.005	0.87	1.14
*Level of education*								
Read and write	0.312	0.494	0.029	0.633	0.527	− 0.658,1.282	0.51	1.96
Primary school	0.757	0.445	0.089	1.702	0.09	− 0.117,1.632	0.38	2.63
Secondary school	1.236	0.431	0.183	2.869	0.004	0.389,2.083	0.26	3.87
College/ University	1.873	0.436	0.296	4.297	**0.000**	1.017,2.730	0.22	4.56
*Know someone diagnosed with stroke (Yes)*	3.64	0.285	0.486	12.7	**0.000**	3.079,4.200	0.72	1.39

## Discussion

The focus of this study was to describe knowledge of stroke risk factors and warning symptoms with special emphasis on factors associated with it. In this study, the mean score of knowledge of stroke risk factors was 67.2 ± 17.1% and the mean score of knowledge of stroke warning symptoms was 63.9 ± 15.4%. Higher educational level, knowing someone diagnosed with stroke, and living with others were independently associated with knowledge of stroke risk factors. Younger age, higher educational level, and knowing someone diagnosed with stroke were independently associated with knowledge of warning symptoms of a stroke.

The participants in this study achieved a modest mean score of knowledge of stroke risk factors. The majority of the participants in our study reported hypertension as a risk factor for stroke, followed by obesity, stress, smoking, and physical inactivity, which is consistent with previous studies conducted in Nigeria, Luxemburg, Beirut, Morocco, and Thailand [34–38]. However, the result of this study is different from that of a study previously conducted in Bahir Dar in which the majority of the participants could not identify at least one risk factor of stroke [33]. This discrepancy may be related to the difference in the study population and source of information that could limit access to information. Our study samples are patients with type 2 diabetes and most of them reported getting stroke-related information from friends and relatives. Though most of our study samples could identify stroke risk factors which previous epidemiological studies have revealed [10, 17, 18], the overall mean score of knowledge of stroke risk factors is lower compared to other studies conducted in western countries. This implies the need to disseminate health information to improve the knowledge of stroke risk factors among adults with type 2 diabetes in the study area.

Similar to previous studies conducted in Beirut, Morocco, and India [36, 37, 39, 40], our findings revealed that type 2 diabetes patients with higher educational levels and those who know someone with diagnosed stroke have better knowledge of stroke risk factors and warning symptoms. Our study also found that type 2 diabetes patients who live with other people have better knowledge of stroke risk factors. These corroborate with the scientific shreds of evidence that explain people with higher educational attainment [41, 42] and who live within the social network [43, 44] often have better knowledge of risk factors and symptoms of chronic illnesses. This implies that since they poorly recognize stroke risk factors and warning symptoms type 2 diabetes patients with lower educational attainment could be more susceptible to developing stroke and delay in help-seeking. Corollary, it is essential to pay special attention to type 2 diabetes patients with low educational levels and who live alone when designing educational and social support programs aimed at improving their knowledge of stroke risk factors.

Our study indicates that the majority of the participants were able to identify symptoms of stroke such as trouble in body coordination, sudden unilateral body weakness, and trouble walking. unlike that of a previous study conducted in Bahir Dar [33], The discrepancy between the present study and the Bahir Dar study findings may be related to the difference in the geographic location of the sample and the health care provision that potentially cause variation in access to health information and health education. But, the results of our study agree with that of previous studies in which the majority of participants identified some of the established stroke symptoms [35, 45, 46]. Nonetheless, our study results indicate the need to put in place educational interventions to improve the overall knowledge level of the warning symptoms of stroke among adults with type 2 diabetes.

Unlike the previous studies that reported the major source of stroke information as television and radio [35, 39] and health care professionals [38], our study showed that friends and relatives are the main sources of stroke information. The discrepancy may be related to the difference in the study population and setting. Our study informs the importance of disseminating stroke-related information not only to type 2 diabetes patients but also to their family members and friends. This will have a dual effect in that the information has an important use value both for the families or relatives and the patients. The families or relatives can use the information provided for themselves and at the times relay it to their members who live with type 2 diabetes. Therefore, the health care team should consider the involvement of families or relatives when teaching type 2 diabetes patients about stroke prevention and other self-management issues.

## Limitation

The findings of our study are limited to type 2 diabetic patients attending government-managed hospitals. These findings may not represent economically better-off diabetes patients who attend private hospitals for their follow-up care. The other limitation is related to the cross-sectional nature of the design which does not lend itself to determining causal relationships between variables under investigation. The quantitative nature of the study using structured questions with fixed response options such as ‘yes’ or ‘no’ did not allow us to probe the reason why the participants hold a particular viewpoint. Thus, future qualitative study with open-ended questions is warranted.

## Conclusion

The mean score of knowledge of stroke risk factors and warning symptoms was 67.2% and 63.9%, respectively. Participants reported hypertension and obesity as the main stroke risk factors while reporting trouble coordination and unilateral body weakness as warning symptoms. Younger age, higher educational level, know someone diagnosed with stroke, and living with others emerged as independent predictors of stroke risk factors and warning symptoms. Therefore, future educational interventions should target type 2 diabetes adults with advancing age and the involvement of their family members to improve knowledge of stroke risk factors and warning symptoms.

### Recommendation

For health professionals, it is better if they form a group of patients with stroke and type 2 DM and discuss stroke risk factors and warning symptoms during their follow-up appointments so that they can easily share experiences. And it’s better if they disseminate information regarding stroke via health education sessions, and posters since this will benefit the community at large. For researchers to undertake other community-based and multi-centered studies on stroke risk factors and warning symptoms which will provide further information. Finally, it would be beneficial for the community if they accomplish a higher level of education since education has an impact on knowledge regarding stroke.

## Supplementary Information

**Additional file 1**. Supplementary figures and table.

## Data Availability

The dataset generated and analyzed during this study is not publicly available to maintain data security but is available from the corresponding author on reasonable request.

## Abbreviations

CVD: Cardiovascular disease
BSc: Bachelor of Science
GDB: Global burden of disease
SPSS: Statistical package for social sciences.
