# Supplementary material for: Knowledge of stroke risk factors and warning symptoms among adults with type 2 diabetes in Addis Ababa, Ethiopia, 2021: an institution-Based cross-sectional study
Source: BMC Cardiovasc Disord. 2023 Jan 16;23:21. doi: 10.1186/s12872-022-03031-8 (PMC9841697; doi:10.1186/s12872-022-03031-8)
Supplement: Supplementary file 1 — Additional file 1. Supplementary figures and table. [file 12872_2022_3031_MOESM1_ESM.pdf]

Supplementary figure and table legends

Figure S 1: linear regression analysis requires all variables to be normally distributed. This assumption can best be checked with histogram. Figure S1 Showing normal distribution of residuals using histogram.

Figure S 2: Normal probability plot assessing how closely the two data sets (distribution of standard residuals from the model against the residuals associated with a normal probablity graph) agree. Figure S2 illustrates P-P plot showing residuals that follows a normal distribution since it follows axis of the chart.

Figure S 3: Another assumptions of linear regression analysis is homoscedasticity. The scatter plot is a good way to check whether the data are homoscedastic. As it is illustrated in the Figure S 3, there is no clear pattern in the distribution. So, the data are homoscedastic.

Table S 1: Linear regression assumes that there is little or no multicollinearity in the data. It is best tested by tolerance (> 0.1) and VIF (< 10). As it is illustrated in the Table S1 there is no multicollinearity in the data.

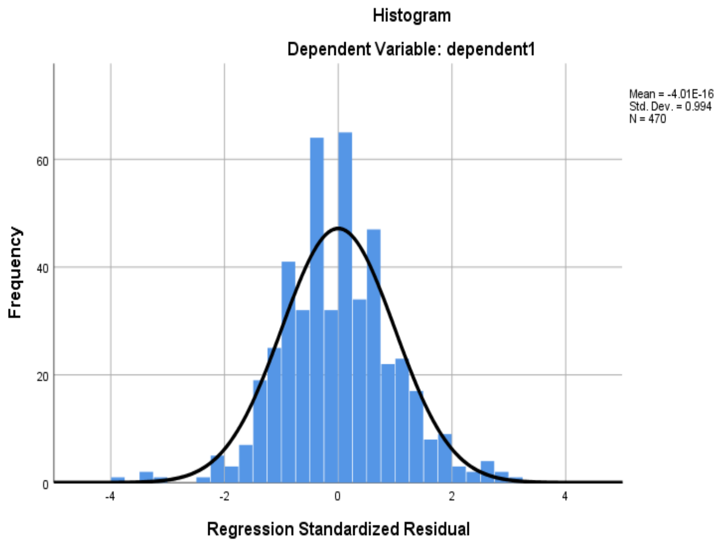

Supplementary figure 1: Showing normal distribution of residuals using histogram

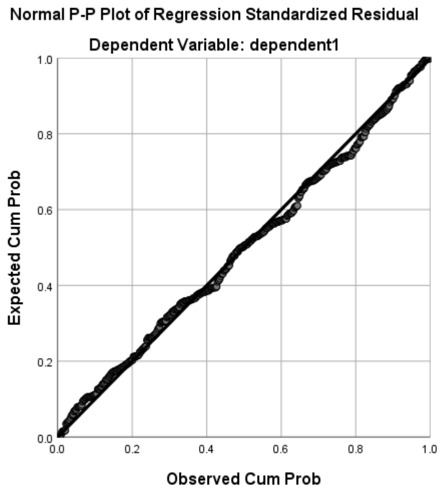

Supplementary figure 2: P-P plot showing residuals that follows a normal distribution

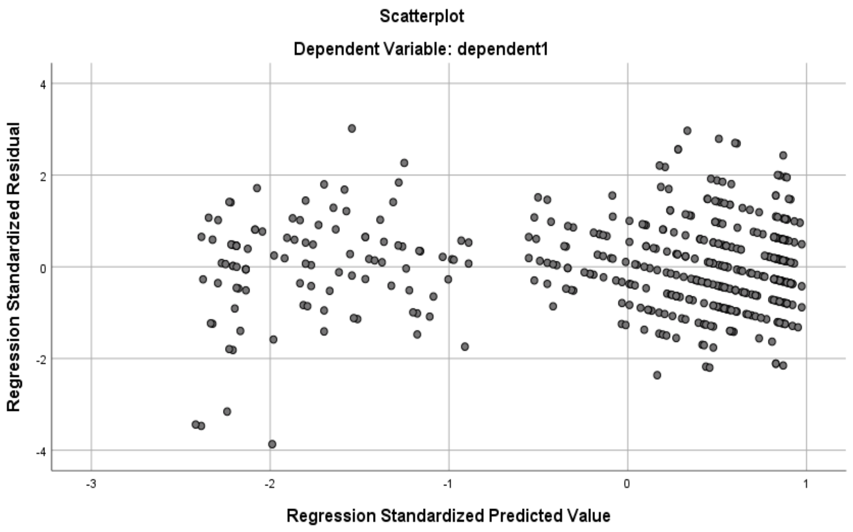

Supplementary figure 3: Scatter plot showing homoscedasticity of the data

Supplementary table 1: Showing collinearity statistics of data including both tolerance and VIP

**Variable**	**Collinearity statistics**	
	**Tolerance**	**VIF**
**Age**	0.87	1.14
**Level of education**		
Read and write	0.51	1.96
Primary school	0.38	2.63
Secondary school	0.26	3.87
College/ University	0.22	4.56
**Know someone diagnosed with stroke**	0.72	1.39
